# Polymer-assisted intratumoral delivery of ethanol: Preclinical investigation of safety and efficacy in a murine breast cancer model

**DOI:** 10.1371/journal.pone.0234535

**Published:** 2021-01-28

**Authors:** Corrine Nief, Robert Morhard, Erika Chelales, Daniel Adrianzen Alvarez, Ioanna Bourla BS, Christopher T. Lam, Alan A. Sag, Brian T. Crouch, Jenna L. Mueller, David Katz, Mark W. Dewhirst, Jeffrey I. Everitt, Nirmala Ramanujam

**Affiliations:** 1 Department of Biomedical Engineering, Duke University, Durham, North Carolina, United States of America; 2 Department of Interventional Radiology, Duke University School of Medicine, Durham, North Carolina, United States of America; 3 The Preston Robert Tisch Brain Tumor Center, Duke University School of Medicine, Durham, North Carolina, United States of America; 4 Department of Obstetrics and Gynecology, Duke University, Durham, North Carolina, United States of America; 5 Department of Radiation Oncology, Duke University School of Medicine, Durham, North Carolina, United States of America; 6 Department of Pathology, Duke University, Durham, North Carolina, United States of America; 7 Department of Pharmacology and Cancer Biology, Duke University, Durham, North Carolina, United States of America; 8 Duke Global Health Institute, Duke University, Durham, North Carolina, United States of America; University of Washington School of Medicine, UNITED STATES

## Abstract

Focal tumor ablation with ethanol could provide benefits in low-resource settings because of its low overall cost, minimal imaging technology requirements, and acceptable clinical outcomes. Unfortunately, ethanol ablation is not commonly utilized because of a lack of predictability of the ablation zone, caused by inefficient retention of ethanol at the injection site. To create a predictable zone of ablation, we have developed a polymer-assisted ablation method using ethyl cellulose (EC) mixed with ethanol. EC is ethanol-soluble and water-insoluble, allowing for EC-ethanol to be injected as a liquid and precipitate into a solid, occluding the leakage of ethanol upon contact with tissue. The aims of this study were to compare the 1) safety, 2) release kinetics, 3) spatial distribution, 4) necrotic volume, and 5) overall survival of EC-ethanol to conventional ethanol ablation in a murine breast tumor model. Non-target tissue damage was monitored through localized adverse events recording, ethanol release kinetics with Raman spectroscopy, injectate distribution with *in vivo* imaging, target-tissue necrosis with NADH-diaphorase staining, and overall survival by proxy of tumor growth. EC-ethanol exhibited decreased localized adverse events, a slowing of the release rate of ethanol, more compact injection zones, 5-fold increase in target-tissue necrosis, and longer overall survival rates compared to the same volume of pure ethanol. A single 150 μL dose of 6% EC-ethanol achieved a similar survival probability rates to six daily 50 μL doses of pure ethanol used to simulate a slow-release of ethanol over 6 days. Taken together, these results demonstrate that EC-ethanol is safer and more effective than ethanol alone for ablating tumors.

## Introduction

Breast cancer is still the leading cause of cancer related mortality for women globally [1]. Chemotherapy, surgery, and radiation therapy are foundational for breast cancer treatment; however, reliable access to surgery and radiation is significantly hindered in low- and middle-income countries (LMICs) [2, 3]. Tumor ablation is an alternative local therapy which uses heat, ice, or cytotoxic chemicals to produce focal destruction of tissue non-invasively. Ablation is typically performed when surgical excision of the tissue is impractical, inaccessible, or dangerous for the patient. Oligometastatic breast cancer lesions in the liver, lungs, and bones can be ablated to achieve local control or reduce pain [4]. Historically, the use of ablation for oligometastatic breast cancer has been controversial [5, 6]; although, more recently the ablation of breast cancer lesions in the liver has been shown to extend progression free survival for patients with advanced disease [7, 8]. Ablation has yet to be accepted as a first-line treatment for primary breast tumors, however, its use for control of metastatic breast cancer warrants further study as a primary tumor therapy for breast cancer in low-resource settings where traditional therapies, like surgery and radiation therapy, are unavailable.

Ethanol ablation, or percutaneous ethanol injection (PEI), consists of simply injecting pure ethanol into lesions and is the cheapest and most readily available ablation modality. Therefore, PEI is an attractive ablative strategy in LMICs as it can be implemented with commonly used, easily sourced medical supplies. Ethanol kills tumors by causing coagulative necrosis upon contact with tissue [5, 9, 10]. Ablation of small focal lesions with an injection of ethanol visualized with ultrasound is a well-accepted technique because it is cost-effective and has acceptable clinical outcomes for treatment of a variety of tumors [11]. Ethanol ablation has been used as an alternative to radiofrequency ablation for small hepatocellular carcinomas in cirrhotic patients because it is less expensive and less time-consuming with a similar 5-year survival rate for small lesions [10, 12]. More recently, ethanol ablation has been used successfully for palliation of osteolytic bone metastases [4, 5, 9, 13]. However, current implementations of PEI are limited by unpredictable control of ethanol resulting in ethanol leakage away from the injection site and incomplete tumor necrosis [14, 15]. In fact, PEI frequently requires multiple sessions to achieve local control, especially for large lesions [16, 17]. For these reasons, microwave ablation and cryoablation have gained favor in settings where they are available [18]. Thermal ablation requires hard to access resources including CO_2_ cylinders for cryotherapy, expensive specialized machinery for microwave ablation, and a consistent power supply, limiting use of thermal ablation in LMICs.

To overcome the limitations of traditional PEI we incorporated the phase-changing polymer ethyl-cellulose (EC). EC is an ethanol-soluble, water-insoluble polysaccharide commonly used as a coating for medical pills and Generally Regarded as Safe (GRAS) by the Food and Drug Administration. EC powder was mixed into the ethanol solution in order to increase the viscosity of the ethanol and limit the spread of ethanol out of the tumor. EC-ethanol’s unique physical properties have been safely utilized clinically to treat venous malformations [19, 20] and herniated discs [21]. We previously optimized an injection method for EC-ethanol in *ex vivo* hepatic tissue, demonstrating that slow infusion rates improve EC-ethanol depot formation [22]. The addition of EC to ethanol ablation was previously shown to eliminate hamster cheek-pouch tumors monitored over 7 days [23], and chemically-induced breast tumors in rats monitored over 30 days [24]. However, the impact of EC on intratumoral delivery and anti-tumor activity has yet to be characterized *in vivo* and EC-ethanol has yet to be assessed for treatment of invasive breast cancers, a leading cause of cancer mortality globally [2, 3].

In this study, we build on our previous work to demonstrate for the first time that the addition of EC to the ethanol injectate improves ethanol retention within the tumor, decreases ethanol diffusion, reduces incidence of adverse events, and improves overall survival in a murine model of breast cancer. Unlike our previous studies, which focused on short term reductions in tumor volume in a head and neck cancer model and characterized EC-ethanol injections in *ex vivo* liver tissue, this study reports the first use of EC-ethanol ablation in invasive breast cancers to induce local necrosis and increase overall survival. Raman spectroscopy and fluorescence imaging were utilized to monitor how EC affects ethanol diffusion through tumor tissue, as ethanol has a distinct Raman spectra [25, 26], and the *in vivo* spatial distribution of EC-ethanol using fluorescein, respectively. Ablation efficacy was validated with viability staining of tumors following ablation to quantify necrosis, as well as long-term monitoring of the tumor volume and overall survival after EC-ethanol ablation. This study lays the groundwork for using EC ethanol as an alternative treatment for primary breast tumors in LMICs where traditional frontline treatments like surgery and radiation therapy remain out of reach for billions of women.

## Materials and methods

### 67NR cell culture and tumor implantation in mice

Subcutaneous flank tumors were established in 6-8-week-old female nu/nu mice (Duke University CCIF) or BALB/c mice (Charles River Labs) through injection of 5x10^5^ 67NR murine luminal breast cancer cells in 100 μL of serum-free RPMI (VWR). Murine breast cell line 67NR was provided by the Dr. Fred R. Miller laboratory (Karmanos Cancer Institute, Detroit, MI) through Dr. Inna Serganova and Dr. Jason Koucher (Memorial Sloan Kettering Cancer Center, New York, NY). 67NR cells are locally invasive but non-metastatic, previously characterized by CJ Aslakson and FR Miller. The 67NR tumors were verified with CK8/18 staining performed by the Dr. Robert Cardiff laboratory at University of California Davis. Mycoplasma testing was performed by the Duke Cell Culture Facility. 67NR cells were cultured in RPMI with 5% FBS, 2 mM L-glutamine, penicillin (100 units/ml), and streptomycin (100 Mg/mL). Mice were euthanized when tumors reached 1500 mm^3^ or body weight dropped 15% below baseline. Tumors were excised post-euthanasia, flash frozen, and processed by the Duke Substrate Core (Duke University, Durham, NC).

### EC-ethanol injections

Anhydrous ethanol was obtained from Koptetc (King of Prussia, NJ). USP grade Ethyl cellulose was purchased from Sigma Aldrich (SKU: 1265504). Fluorescein (free acid) 95% purity was purchased from Sigma Aldrich (SKU: F2456). All experiments used 2.5% w/w of fluorescein mixed in ethanol. The stability of EC polymer is well documented [27], however the rate of EC degradation in pure ethanol has yet to be quantified. To limit possibility of chemical modification of EC polymers, 6% EC-ethanol solution was mixed without heat no more than 24 h before injection. All mixtures were mixed with an ethanol safe stir-bar in ethanol-safe containers. All injections were performed using 27-gauge needles with manual needle placement in the center of the tumor. Injections were performed at a fixed rate of 1 mL/h with a syringe pump unless otherwise specified.

### Adverse events recording in mice

All animal work was performed under approved protocol A160-18-07, approved by the Duke University Institutional Animal Care and Use Committee (IACUC). All experiments were performed in accordance with relevant animal welfare guidelines and regulations. Mice were group housed in cages of 5 with standard chow and water ad libitum. Mice were enriched with supplemental housing and bedding. Isoflurane (1–3% v/v) anesthesia was used for all injections. Mice were euthanized by carbon dioxide inhalation and bilateral thoracotomy. Murine 67NR tumors were grown to 5–10 mm diameter in the flank of 98 nu/nu mice. A dose escalation study was performed using EC-ethanol injection volumes between 2–16 mL/kg. Once a dose was demonstrated to be non-lethal for a majority of the mice, the dose was increased with a starting dose of 2 mL/kg. Death within 24 h of treatment qualified as lethality. Doses above 6 mL/kg were found to be unsafe (lethal for a majority), so testing above 6 mL/kg was limited to a small number of mice. For comparison, 16 mice were given a single intratumoral injection of 6 mL/kg of ethanol and fluorescein alone, 5 mice received 50 μL of ethanol daily for 6 days referred to as the “repeated ethanol” schedule to simulate a slow release of ethanol, and 18 mice received no injection. One mouse died hours after a 4 mL/kg dose; however, the mouse was also under isoflurane anesthesia for a prolonged time (1 hour, 3%). Thus, considerations were made to limit isoflurane exposure after this adverse event. No other changes in systemic adverse events was noted for doses below 6 mL/kg. Mice were monitored for adverse events for a minimum of 14 days and up to 35 days (dictated by tumor growth) to observe any delayed effects after treatment. Greater than 200 breaths per minute qualified as respiratory distress. Limping or dragging of the tumor-bearing leg at any point indicated mobility impairment. Swelling and redness at any time after injection was noted as inflammation/edema. Bruising or bleeding of the tumor-bearing leg was noted as bleeding. Ulceration was defined as a scab on the skin over the tumor persisting for more than 3 days. A drop of 15% or greater of the baseline weight at any time after treatment was recorded as a severe loss in body weight. Because the tumors in this study were on the flank, mobility impairment, inflammation, edema, and ulceration of the tumor-bearing foot were considered localized adverse events. Lethality, respiratory distress, and loss in body weight were considered systemic adverse events.

### Quantification of ethanol diffusion coefficient using Raman spectroscopy

The diffusion coefficient of ethanol through tumor tissue was measured using a Raman spectroscopy assay coupled with a deterministic mathematical model of ethanol diffusion. Tumor specimens (n = 6) were cut longitudinally and trimmed to fit 12 mm Snapwell inserts containing porous polycarbonate membranes (Corning-Costar^®^, New York, NY). Each snap well was placed in a custom-built chamber filled with 600 μL of ethanol to enable ethanol diffusion through the membrane and upwards into the tumor (Fig 2). A custom-built confocal Raman spectroscope with a 785 nm excitation laser diode (LD785-SH300, Thorlabs Inc., Newton, NJ) was used to capture Raman spectra at the surface of the tumor [28]. The least squares fit was performed in MATLAB (MathWorks, Natick, MA) to extract the relative contribution of ethanol and tumor tissue to the measured Raman spectra at the tumor surface, using a previously validated method [28–30].

To compute the diffusion coefficient of ethanol, a deterministic two-compartment transport model was used that recapitulates the Raman assay, using Fick’s law of diffusion to characterize ethanol diffusion from an ethanol compartment at the bottom, to the tumor at the top (S1 Fig). An analogous model was outlined previously in and was applied to estimate the diffusion coefficients of drugs in biological tissues [30]. Here, we assumed that the partition coefficient between the ethanol and tumor compartments (ΦET) was 1 due to the high porosity of the polycarbonate membrane, which is fully permeable to small molecules like ethanol. The diffusion coefficient of pure ethanol is 10^−5^ cm^2^/s, and the height of the ethanol compartment, hE, was 1.5 cm. The height of each tumor sample, hT, was measured using a micrometer. The two unknowns, the diffusion coefficient of ethanol in the tumor (DT) and the first-order loss term that accounts for ethanol evaporation from the tumor (KEV) were computed and optimized by fitting the predicted concentration of ethanol at the surface of the tumor to the measurements obtained using Raman spectroscopy.

### Whole-body fluorescent imaging

Mice were imaged using a whole-body *in vivo* IVIS Lumina imaging system (Perkin Elmer) in the prone position, 30 minutes after injection with 6% EC-ethanol or pure ethanol (n = 5) to allow for injectate diffusion. Images were acquired with excitation at 430 nm, emission at 520 nm, and an exposure time of 1 second. Mice with 5 mm 67NR flank tumors (n = 5) were given an injection of 6 mL/kg of either 6% EC-ethanol or pure ethanol. Pixels with a radiant efficiency of 0.5x10^10^ or higher we selected to represent the injection area. Area calculations and compactness calculations were performed using standard image processing tools in MATLAB (MathWorks).

### Imaging with a hand-held fluorescence microscope

For *ex vivo* imaging, 6% EC-ethanol was injected into ~1000 mm^3^ tumors. Tumors were injected with 150 μL of either pure ethanol (n = 10), 3% EC-ethanol (n = 10), or 6% EC-ethanol (n = 20) at 1 mL/h. A control image of a tumor without any injection was acquired to account for tumor autofluorescence. Tumors were flash frozen and cross-sectioned centrally for imaging at a 10 mm working distance. Tumors were imaged using a fluorescence microscope with excitation at 480 nm and an emission bandpass filter at 520±15 nm. Pixels with >80% saturation in the green channel were counted as the area of fluorescence. Pixel dimensions were converted to an area using a digital ruler to scale each set of photos, and the radius r_1_ was computed. The fluorescent volume V_1_ (calculated using r_1_) was calculated assuming the fluorescent image was taken at the center of a spherically distributed injection.

### Histology and immunohistochemistry

For viability staining, a 5 μm section was taken serially every 2 mm throughout the tumor and stained with NADH-diaphorase to quantify necrosis. NADH-diaphorase staining was performed using Nitrotetrazolium Blue Chloride (Sigma-Aldrich) and B-Nicotinamide Adenine Dinucleotide (Sigma-Aldrich). The region of interest (ROI) was selected manually from a digital scan at 5x magnification to include only non-viable cellular regions. Adjacent H&E slides were used to confirm tumor regions. Necrotic volume was calculated by the Reimann Sum: $V=\sum_{i=1}^{n}A(x_{i})\Delta x$, where volume (V) is equal to the summation of area (A) from 1 to n times the distance between each measurement (x). In this case, x = 2 mm.

### Tumor growth and survival

For tumor growth and survival studies, mice received 6% EC-ethanol injection four days after cell inoculation, when tumors were palpable and ~50 μL in volume. Mice were assigned to receive either no treatment (n = 8), a single injection of pure ethanol at 6 mL/kg (n = 6), repeated injections of pure ethanol at 50 μL/day for 6 days (n = 5), or a single injection of 6% EC-ethanol at 6 mL/kg (n = 10). Mice were euthanized when the tumor reached 2000 mm^3^ (2 cm^3^). Time to a tumor volume of 1500 mm^3^ was used as a surrogate for survival time. If a humane endpoint was met before the maximum tumor burden, the humane endpoint was recorded as an adverse event. Tumor length (L) and width (W) was measured using calipers, and volume (V) was calculated using V = (W^2^ × L)/2. Average tumor volume does not include deceased mice.

### Statistical analysis

Statistical analyses were performed in MATLAB using built-in functions. Rates of adverse events were assessed between subjects with a Chi Squared test with a confidence level of 95% with a degree of freedom of 1. Outliers were detected with a Grubbs Test. No outliers were found to be excluded. Two-tailed ANOVA testing with unequal variance was performed on all groups between each other and controls with a confidence level of 95%. Post-hoc multiple comparisons were performed using Tukey’s HSD test. Survival curves were quantified using Kaplan-Meier analysis, and a log-rank test was performed to determine the significance of a *P*-value less than 0.05 with a confidence level of 95%.

## Results

### Adverse events compared to pure ethanol and EC-ethanol dose selection

**Fig 1:**
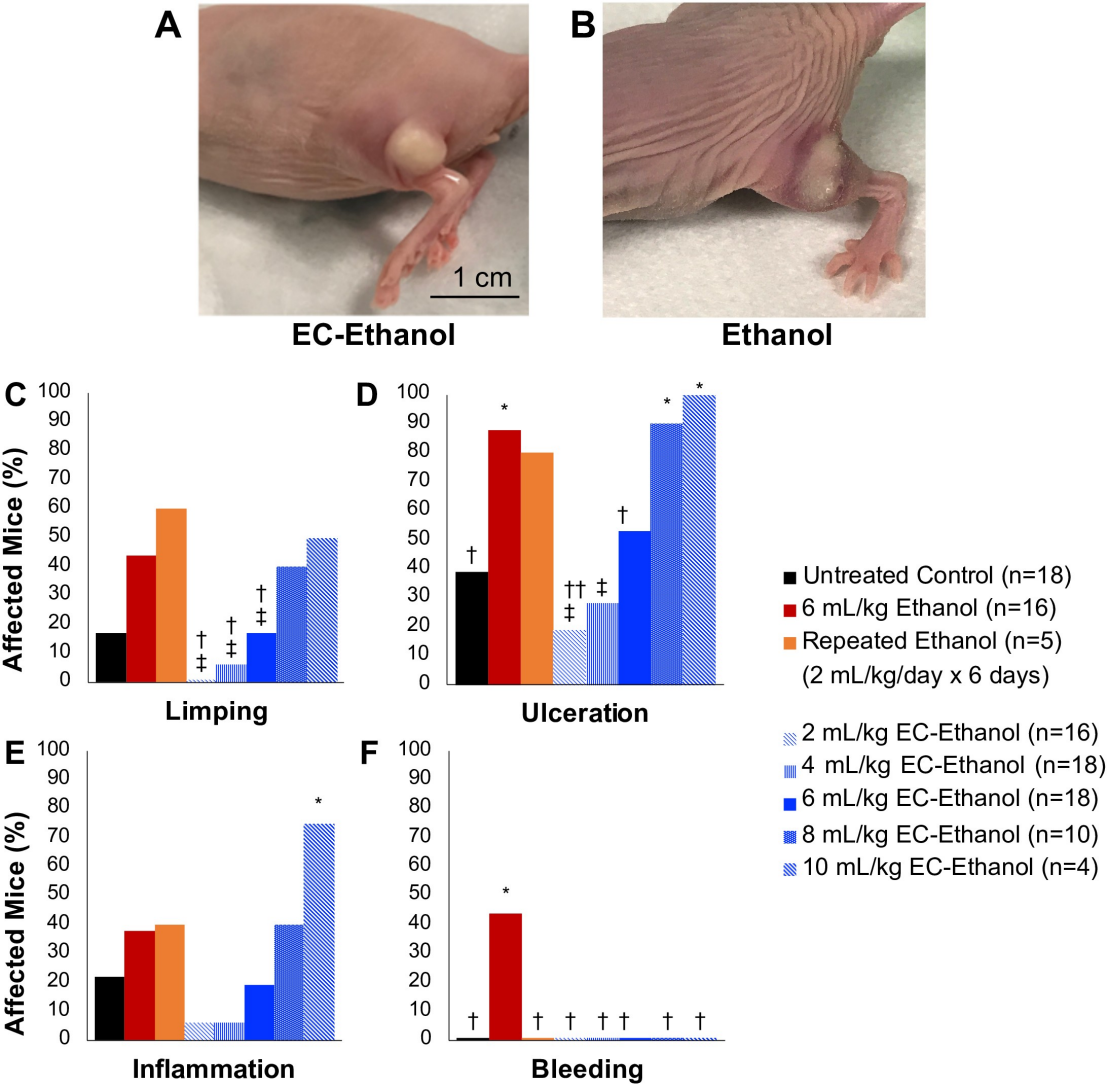
Localized adverse events after EC-ethanol compared to pure ethanol. Mouse flank tumor after injection of (**A**) 6% EC-ethanol and (**B**) ethanol. Rates of (**C**) limping, (**D**) tumor ulceration, (**E**) inflammation, and (**F**) subdermal bleeding after each treatment protocol. Athymic nude mice with 67NR tumors grown on their flanks were injected at a rate of 1 mL/hr when tumors were approximately 50 mm^3^ in volume. Chi squared test significance of *P*<0.05 is indicated with (*) asterisk compared to untreated group, (†) dagger compared to 6 mL/kg ethanol group, (‡) double dagger compared to repeated 6 x 2 mL/kg/day ethanol group.

We investigated whether using EC-ethanol would impact the rates of adverse effects compared to pure ethanol injections (recorded in S1 Table). Mice with 67NR flank tumors received a single intra-tumoral injection of different volumes of 6% EC-ethanol (2–10 mL/kg), 4 days after tumor inoculation when tumors were approximately 50 μL in volume. A dose of 6 mL/kg of EC-ethanol was identified as the maximum tolerable dose (MTD) because it did not cause significantly more systemic adverse than untreated tumor bearing controls (S1 Table). These mice were compared to both mice that received a single intra-tumoral injection of 6 mL/kg (150 μL for average 25 g mouse) of ethanol or a repeat dose of 2 mL/kg (50 μL for average 25 g mouse) of ethanol over 6 days. All injections were delivered at a rate of 1 mL/hr. An untreated tumor bearing group was also included. A mouse was considered to have a localized adverse event if it displayed signs of mobility impairment (limping), subdermal bleeding, inflammation, or ulceration (scabbing) at any time during the two-week monitoring period. The EC-ethanol depot could be visualized grossly in nude mice with flank tumors (Fig 1A and 1B, S2 Fig). Mobility impairment in the form of dragging the tumor-bearing leg or limping was most likely due to muscle or nerve damage in the leg from either ethanol or tumor invasion. EC-ethanol mixtures reduced limping, ulceration, and bleeding, but not inflammation, when compared to the same dose of pure ethanol (Fig 1C–1F). A 6 mL/kg dose of EC-ethanol resulted in less local bleeding, inflammation, and limping than the same dose of pure ethanol, indicating that EC helps limit off target effects of intratumoral ethanol injections.

### EC-ethanol slows ethanol diffusion

**Fig 2:**
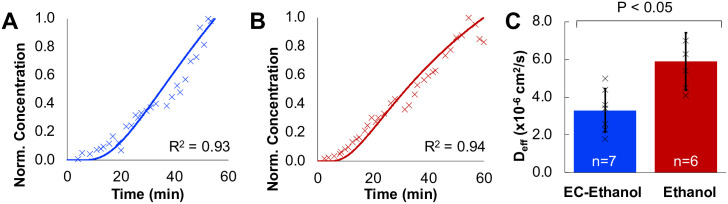
Quantification of diffusion coefficient of EC-ethanol and pure ethanol with Raman spectroscopy. (**A**) Representative ethanol concentration measurements for 6% EC-Ethanol measured with Raman spectroscopy and fit to a diffusion curve for D_eff_ of 1.9x10^-6^ cm^2^/s. (**B**) Representative ethanol concentration for pure ethanol measured with Raman spectroscopy and fit to a diffusion curve for D_eff_ of 5.4x10^-6^ cm^2^/s. (**C**) The effective diffusion coefficient was slower when using EC-ethanol (n = 7) compared to pure ethanol (n = 6). Bars indicate SD. *P<0.05 using Tukey’s HSD test.

In order to investigate the mechanism for improved safety of EC-ethanol over ethanol, the release kinetics of ethanol through tissue were investigated using Raman Spectroscopy. The tissue-depot interface was modeled as shown in S1 Fig. EC was hypothesized to reduce off-target leakage by slowing the release of ethanol from the injected depot site. The relative concentration of ethanol over time a sample was measured with Confocal Raman Spectroscopy as ethanol diffused through thick (5–10 mm) sections of 67NR tumors. The change in ethanol concentration over time was used to fit a model of molecule transport in a two-compartment model. The effective diffusion coefficient (D_eff_) of a molecule represents the effective transport rate at which molecules move from one compartment to the other. D_eff_ can be approximated from the concentration over time recordings (Fig 2A and 2B) as in previously validated algorithms [28–30]. The D_eff_ of ethanol of 6%EC-ethanol was approximately half that of pure ethanol (3.3 ± 1.5 x 10^−6^ cm^2^/s vs. 5.9 ± 1.2 x 10^−6^ cm^2^/s; *P*<0.05; n = 7,6) (Fig 2C). Taken together, these results demonstrate that addition of EC to ethanol significantly slows the release of ethanol through tumor tissue.

### EC-ethanol delivery is more compact compared to pure ethanol

**Fig 3:**
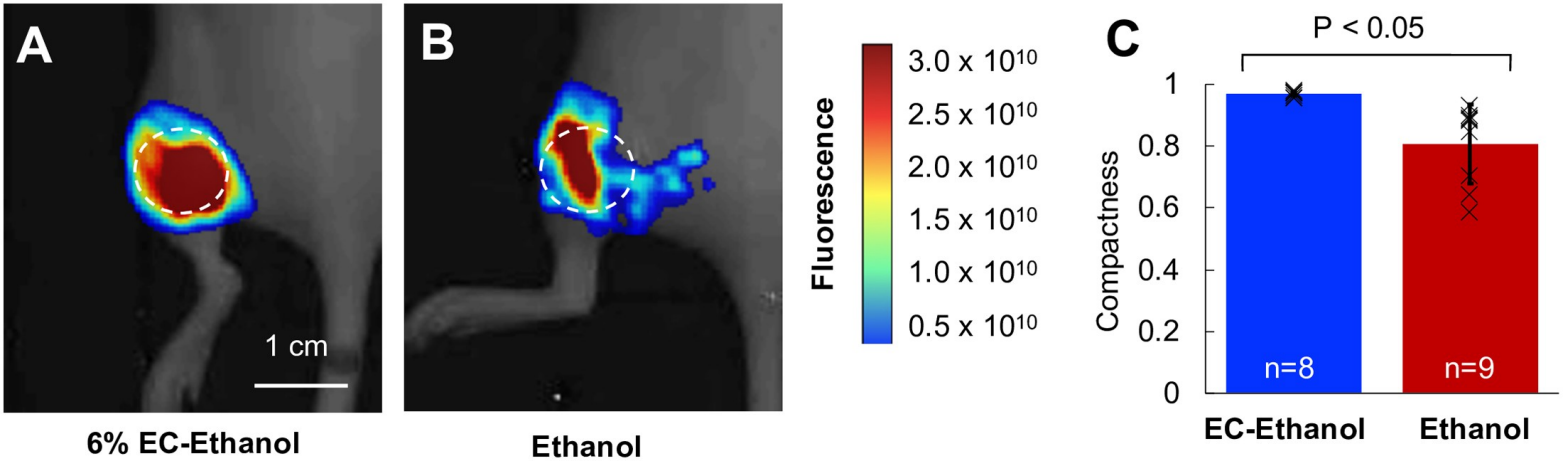
Injection of EC-ethanol yields a more compact injection zone than pure ethanol. (**A, B**) *In vivo* images showing the fluorescent volume following a single injection of 150 μL of 6%EC-ethanol or pure ethanol. Images were acquired 30 min after injection. The dashed circle indicates the approximate tumor location. **(C**) The compactness of the injectate was greater for EC-ethanol (n = 8) than for pure ethanol (n = 9).

To demonstrate that the slower diffusion of EC-ethanol influences the local distribution of ethanol, the localization of EC-ethanol and ethanol-only injections were quantified. Both EC-ethanol and pure ethanol were mixed with the fluorescent dye, fluorescein (2.5% w/w) to enable *in vivo* tracking of the injectate. Small, 50 mm^3^ 67NR tumors were injected with 150 μL at 1 mL/hr to force overflow from the tumor and monitor the event of tumor leakage. Nude mice were used to limit autofluorescence from fur. Whole-body imaging was performed 30 min after injection (Fig 3A and 3B) using an *in vivo* imaging system (IVIS Lumina, Perkin Elmer. Compactness—defined as 4πA/P^2^, where A and P are the area and perimeter of the fluorescent region, respectively—was calculated using MATLAB. A perfect circle has a compactness of one, and any deviations from a circle will have a compactness that deviates from one. The compactness of the EC-ethanol injectate was significantly greater than that of pure ethanol (0.96 ± 0.01 vs. 0.80 ± 0.13; *P*<0.05; n = 8,9) (Fig 3C), indicating reduced injectate leakage when using the EC-ethanol mixture over pure ethanol.

### EC-ethanol increases intra-tumoral ethanol retention

**Fig 4:**
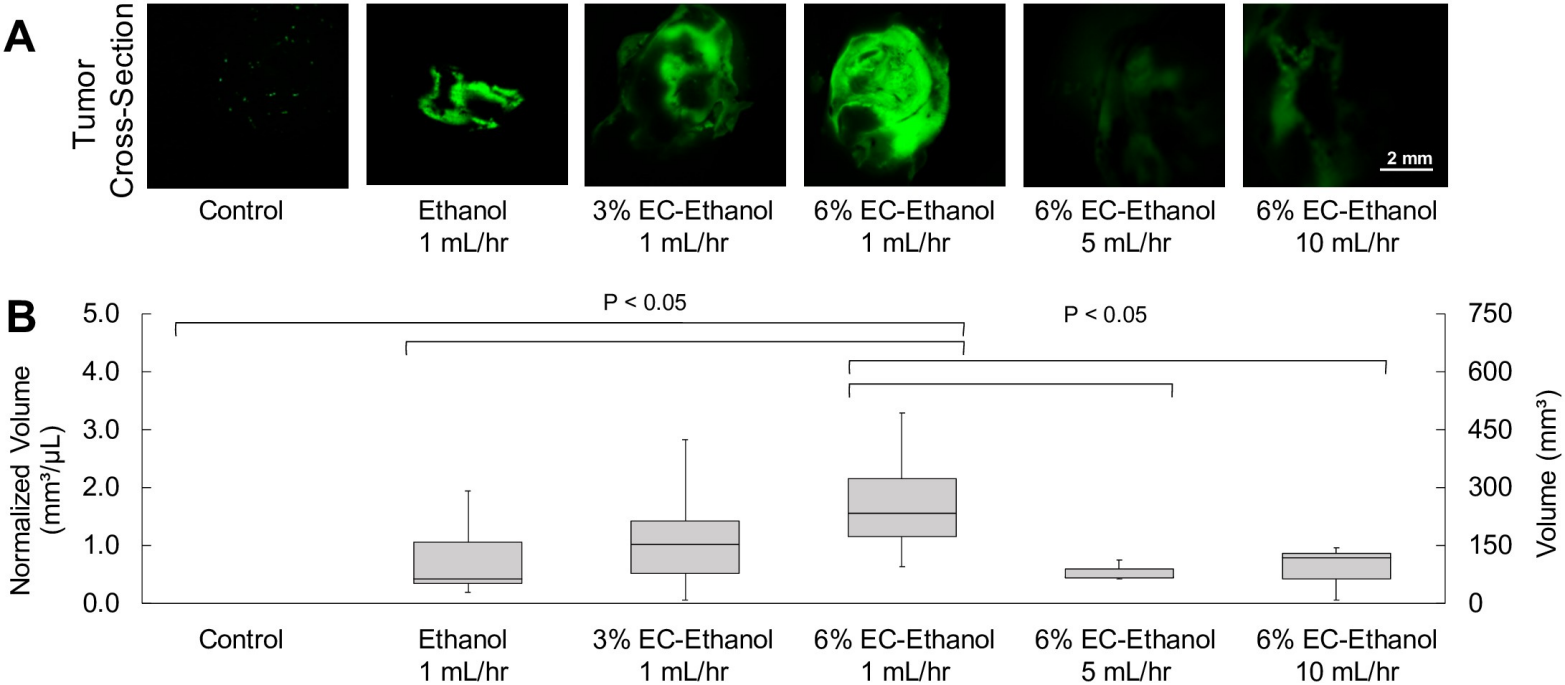
Relationship between EC concentration, flow rate and intra-tumoral ethanol retention. (**A**) Widefield fluorescence microscopy of large (1 cm^3^) 67NR flank tumors cross-sections after 150 μL injections of pure ethanol, 3% EC-ethanol, or 6% EC-ethanol at flow rate of 1 mL/hr and for 6% EC-ethanol at additional flow rates of 5 mL/hr and 10 mL/hr. (**B**) Intra-tumoral retention volume normalized by injection volume of 150 μL.

The effect of relative concentration of EC and injection rate on injectate retention in the tumor were evaluated. A large, 1 cm^3^ tumor volume was selected to replicate clinically relevant tumor sizes while remaining under the ethical tumor burden limit for mice of 2 cm^3^. Nude mice with 1 cm^3^ 67NR flank tumors received a single 150 μL intra-tumoral injection of either pure ethanol, 3% EC-ethanol, or 6% EC-ethanol. Injections of pure ethanol (n = 10), 3% EC-ethanol (n = 10), and 6% EC-ethanol (n = 20) were performed at 1 mL/hr Injections and also at 5 mL/hr (n = 5) and 10 mL/hr (n = 5) for 6% EC-ethanol injections. The contrast agent fluorescein (2.5% w/w) was added to image intra-tumoral injectate retention using fluorescence microscopy. The area of fluorescein was quantified in frozen tumor cross sections immediately after injections as outlined in the methods section. The intra-tumoral fluorescein retention was greater following injection of 6% EC-ethanol than following injection of 3% EC-ethanol or pure ethanol (*P*<0.01; all others n.s.) (Fig 4A and 4B). The intra-tumoral fluorescein retention was also greater when using slower injection flow rates: injection of 6% EC-ethanol at 1 mL/hr yielded a greater fluorescent volume than at 5 or 10 mL/hr (*P*<0.05) (Fig 4A and 4B). The ratio of fluorescent volume to injected volume was 1.85 ± 1.00 for 6% EC-ethanol, which was significantly greater than that for 3% EC-ethanol (1.02 ± 0.75, P<0.05) or pure ethanol (0.74 ± 0.58. P<0.05).

### 6% EC-ethanol results in larger tumor necrotic volumes compared to 3% EC-ethanol or pure ethanol

**Fig 5:**
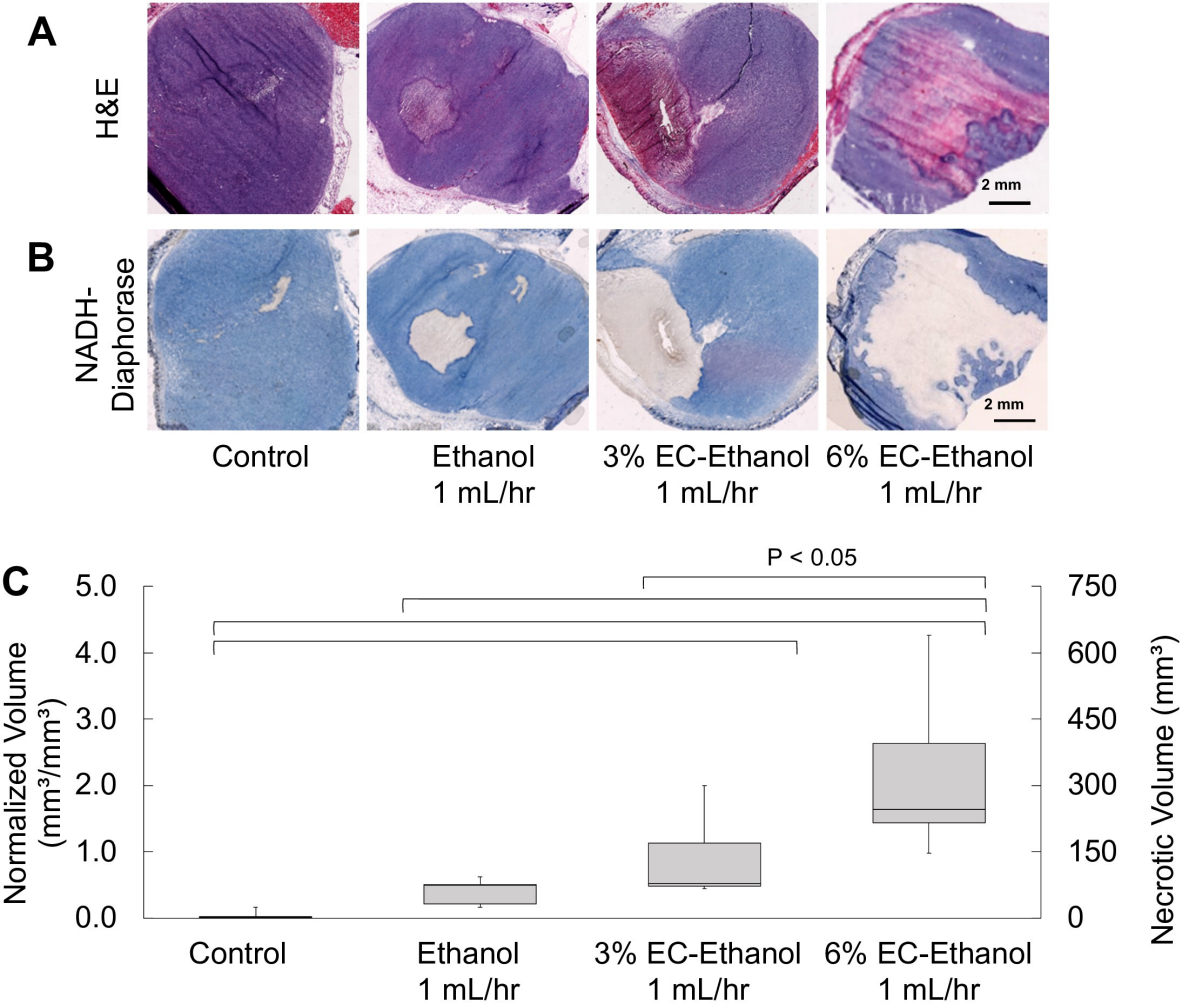
Relationship between EC-ethanol concentration and necrosis. Representative images of (**A**) H&E stained sections and (**B**) adjacent NADH-diaphorase stained sections from frozen blocks. (**C**) Ratio of necrotic volume to injected volume (normalized volume) after injection of 150 μL of pure ethanol at 1 mL/hr (n = 5), 3% EC-ethanol at 1 mL/hr (n = 5), or 6% EC-ethanol at 1 mL/hr (n = 5). * *P*<0.05 using Tukey’s HSD test.

Next, viability staining was performed to confirm that fluorescein retention corresponded to ethanol exposure, and thus necrosis. 67NR tumors were selected because they experience minimal natural tumor necrosis at large volumes, as seen in the untreated mice here. The necrotic volume following EC-ethanol injections was assessed with NADH-diaphorase viability staining. A large (1 cm^3^) tumor volume was selected to replicate clinically relevant tumor sizes while remaining under the ethical tumor burden limit. After grown to approximately 1 cm^3^, 67NR tumors were injected with 150 μL of pure ethanol, 3% EC-ethanol, or 6% EC-ethanol at 1 mL/hr. At 24 hours post treatment, tumors were flash frozen, serially sectioned every 2 mm, and stained with Hematoxylin and Eosin (H&E) to confirm tumor presence (Fig 5A) and NADH-diaphorase to distinguish viable cells (blue) from necrotic cells (white) (Fig 5B). Untreated tumors were included as a control. The necrotic volume following injection of 6% EC-ethanol was 5-fold greater than that of pure ethanol. The average ratio of necrotic volume to injected volume for 6% EC-ethanol was 2.00 ± 0.42, which was significantly greater than 3% EC-ethanol (0.80 ± 0.2) or pure ethanol (0.40 ± 0.09) (Fig 5C). As inducing necrosis is the primary objective of any ablation technique, 6% EC-ethanol is more effective at inducing target tissue necrosis compared to 3% EC-ethanol or pure ethanol.

### EC-ethanol reduces the rate of tumor growth and increases survival rates compared to pure ethanol, obviating the need for repeat injections

**Fig 6:**
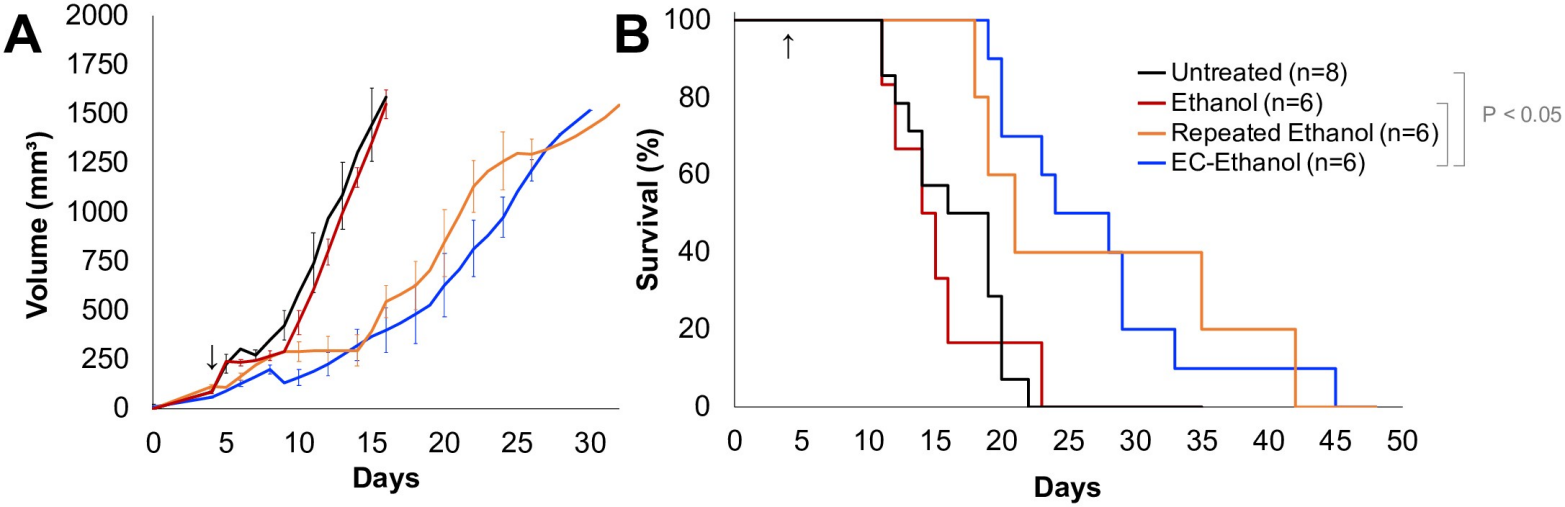
Increased Survival following 6% EC-ethanol Injections Compared to the Same Dose of Pure Ethanol: (E) Average tumor growth for all groups. (F) Kaplan-Meier survival probability. Arrow indicate first day of treatment. Log-rank test on Kaplan-Meier survival curves, pair-wise *P*<0.05, all other pairs n.s.

To quantify long-term therapeutic efficacy, 67NR tumors in BALB/c mice grown to approximately ~50 mm^3^ in volume were randomly assigned to receive either no treatment (n = 8), 150 μL of ethanol (n = 6) or 150 μL of 6% EC-ethanol (n = 6) at a rate of 1 mL/hr. Additionally, a repeated schedule of 50 μL was used for daily injections of ethanol for 6 days, to test the hypothesis that EC-ethanol is functionally similar to a slow release of repeated ethanol injections (n = 6). Tumor size was measured with calipers three times per week. Time to a tumor volume of 1500 mm^3^ was used as a proxy for survival time. Average tumor growth rates are reported in Fig 6A. Survival probability was quantified with Kaplan-Meier curves in Fig 6B. Mice treated with a single ethanol injection did not experience a growth delay compared to untreated controls. Only mice treated with EC-ethanol experienced significantly increased survival rates compared to untreated controls and the same dose of ethanol (Fig 6B). Repeated ethanol injections did not produce significantly greater survival times compared to any group (Fig 6B).

## Discussion

This study reports the first use of EC-ethanol ablation for breast cancers, to date. While previous reports have demonstrated anti-tumor capability of EC-ethanol over a short term (7 days) [23], our study is the first to demonstrate that EC-ethanol reduces adverse events compared to ethanol alone, and that EC-ethanol improves survival rates in mice with breast cancer compared to ethanol alone or untreated controls. Prior studies have demonstrated that EC helps limit ethanol leakage in healthy *ex vivo* liver tissue [22]. Our results build on these studies by demonstrating that EC improves *in vivo* ethanol localization in tumors and slowed ethanol diffusion through tumor tissue, which may account for the increased safety and efficacy. Extra-tumoral leakage of ethanol out of hepatic [31] and thyroid [32, 33] lesions after PEI was found to be proportional to the degree of clinical complications, supporting our findings that the addition of EC to the ethanol injectate increases safety. Finally, the addition of EC to PEI achieved comparable median and overall survival to repeat ethanol-only injections, making PEI with EC potentially feasible for more indications than previously explored.

Cell death due to ethanol is dependent on two factors: the concentration of ethanol and the exposure time. Thus, our goal was to increase the amount of time tumor tissue was exposed to ethanol while limiting ethanol exposure to non-target tissue. We found that slowing the injection rate of the EC-ethanol infusion was advantageous for fluid control, potentially because it allows the tissue to relax and stretch without tearing which would result in aberrant fluid flow. Consistent with our own findings, other reports of intratumoral infusions demonstrate an association between decreased infusion rates and decreases in fluid pressure and thus tissue stress and tearing [34]. Ethanol’s cytotoxic ability is due to the fact ethanol molecules are very-small and polar; thus, tagging ethanol with a radio-opaque or fluorescent probe to monitor its location was deemed impractical for this study as it would affect the transport of ethanol through tissues. 6% EC slowed the effective diffusion of ethanol through tumor tissue as demonstrated with Raman spectroscopy. The intratumoral retention of ethanol was confirmed by assessing the amount of fluorescein dye in the tumor after EC-ethanol-fluorescein injections, and the necrotic volume produced 24 hours later was used to confirm this finding. Injections of 6% EC-ethanol produced a necrotic volume that is 5 times larger than the same dose of pure ethanol. Increased ethanol retention using higher EC concentrations and slower infusion rates ultimately resulted in increased survival times over the same dose of pure ethanol and untreated controls. Notably, survival rates of mice treated with 150 μL 6% EC-ethanol were not significantly different from six 50 μL repeated pure ethanol injections. By adding EC, the number of treatment sessions needed for PEI could be consolidated. We have shown here that a single EC-ethanol can limit tumor growth for aggressive murine 67NR tumors, where ethanol ablation was previously ineffective, requiring multiple injections to slow tumor growth.

While EC-ethanol increased tumor necrosis and survival, no mice lived longer than 45 days after tumor inoculation. There are several potential ways to improve the efficacy of EC-ethanol ablations to increase the number of mice that achieve complete cure. One straightforward approach is to inject in multiple regions throughout the tumor. In this study the needle was placed in the center of the tumor for the duration of the injection, however multiple spatially separated injections may further improve efficacy by providing complete tumor coverage which could be confirmed with ultrasound in larger tumor models. Because the maximum tolerable dose (MTD) given in this study was limited by the body weight of the mouse, larger dose-to-tumor volume ratios could be tested in larger animal models. In humans, larger injection volumes could be used, and the injection rate may need to be faster than 1 mL/hr to deliver large enough doses in a reasonable amount of time. Notably, a 150 μL injection of 6% EC-ethanol resulted in 300 mm^3^ of necrosis which was 5-fold greater than that of pure ethanol alone. Thus, 6% EC-ethanol may enable the ablation of larger lesions than what was previously possible with ethanol alone (~ 2 cm) [17]. While future studies are needed to compare EC-ethanol to standard-of-care treatments, EC-ethanol ablation may enable safe use of ethanol ablation for more indications than previously imagined. Further studies are needed to confirm the clinical feasibility of EC-ethanol for tumors in other locations.

In the future, ablation with EC-ethanol injections could double as a delivery carrier for additional imaging agents or therapeutics, as demonstrated here with fluorescein. More fluorescein was retained in the tumor when EC was included with ethanol injections, thus hydrophobic molecules with similar size and polarity to fluorescein, such as paclitaxel, docetaxel, and tamoxifen, may behave similarly in solution with EC-ethanol. Combining ablation with chemotherapy could potentially create a more robust response compared to either one alone, as has been shown with trans-arterial chemoembolization (TACE) [35]. Additionally, EC-ethanol could be paired with immunomodulatory agents enhance the anti-tumor response to ablation. As EC-ethanol creates necrotic debris like other ablation modalities, there is a potential for adaptive immune system activation through the release of tumor antigens as seen with cryotherapy and thermal ablation [36]. Moreover, the response to immunotherapies is known to be bolstered by the inflammation produced by local tumor ablation [36]. Thus, future studies should investigate the effect that EC-ethanol ablation has on drug delivery and antitumor immunity.

EC-ethanol ablations are ultra-low-cost, allowing their use in low- and high-resource settings alike in the absence of bulky, expensive machinery. With rising inequities in healthcare access, there is a considerable need for the development of easily-accessible options for cancer therapy [37]. For breast cancer, which kills more than 500,000 women each year, access to care is a significant predictor of long-term survival [38–40]. Given that 9 out of 10 people in LMICs lack access to basic surgical care, the most common frontline treatment for breast cancer, there is a critical need for therapies that can serve as surgical alternatives for the estimated 5 billion people without access to essential surgery. While future studies are needed to investigate how EC ethanol compares to surgery, this report demonstrated proof-of-concept that EC-ethanol treatment improves therapeutic efficacy and safety compared to traditional PEI in breast cancer. In the future, EC-ethanol may provide an additional treatment option enabling more patients to access therapy.

## Supporting information

S1 Table Counts of systemic and localized adverse events after ethanol injections. Number of mice affected by local adverse events following a single intratumoral injection of EC-ethanol or pure ethanol.
(PDF)

S1 Fig Raman spectroscopy diffusion quantification. The methodology used to quantify the diffusion coefficient of ethanol in the tumor (DT). The experimental setup used for the Raman spectroscopy assay (left), consists of a tumor laying on a fully permeable membrane that allows seamless diffusion of ethanol upwards into the tumor. The CRS instrument captures the concentration of ethanol at the surface of the tumor over time. A deterministic transport model (right) characterizes ethanol transport and enables optimization of DT via fitting between the Raman measurements and the theoretical predictions.
(PDF)

S2 Fig Representative images of EC-ethanol and ethanol injections in nude mice. Mice after 100 μL injection of either A) EC-ethanol or B) ethanol alone into flank tumors. Nude mice used to show blanching and redness. Images taken approximately 5 minutes after injection.
(PDF)

S1 Graphical Abstract The inclusion of ethylcellulose limits extra-tumoral leakage of ethanol and increases the target-tissue ablation. (PDF)
