# Supplementary material for: Polymer-assisted intratumoral delivery of ethanol: Preclinical investigation of safety and efficacy in a murine breast cancer model
Source: PLoS One. 2021 Jan 28;16(1):e0234535. doi: 10.1371/journal.pone.0234535 (PMC7843014; doi:10.1371/journal.pone.0234535)
Supplement: S1 Table — Number of mice affected by local adverse events following a single intratumoral injection of EC-ethanol or pure ethanol. (PDF) [file pone.0234535.s001.pdf]

Group	Number of Mice	Systemic Adverse Events			Localized Adverse Events			
		Lethality	Respiratory Distress	Loss in Body Weight	Mobility Impairment	Bleeding	Inflammation / Edema	Ulceration
Untreated Control	18	0 (0%)	0 (0%)	0 (0%)	3 (17%)	0 (0%)	4 (22%)	7 (39%)
Ethanol 6 mL/kg	16	0 (0%)	0 (0%)	0 (0%)	7 (44%)	7 (44%)	6 (38%)	14 (88%)
"Repeated Ethanol"	5	0 (0%)	0 (0%)	0 (0%)	3 (60%)	0 (0%)	2 (40%)	4 (80%)
EC-Ethanol 2 mL/kg	16	0 (0%)	0 (0%)	0 (0%)	0 (0%)	0 (0%)	1 (6%)	3 (19%)
EC-Ethanol 4 mL/kg	18	1 (6%)	0 (0%)	1 (6%)	1 (6%)	0 (0%)	1 (6%)	5 (28%)
EC-Ethanol 6 mL/kg	18	0 (0%)	0 (0%)	1 (6%)	3 (17%)	0 (0%)	3 (16%)	9 (50%)
EC-Ethanol 8 mL/kg	10	1 (10%)	3 (30%)	0 (0%)	4 (40%)	0 (0%)	4 (40%)	5 (100%)
EC-Ethanol 10 mL/kg	4	0 (0%)	1 (25%)	0 (0%)	2 (50%)	0 (0%)	3 (75%)	4 (100%)
