# Supplementary material for: Polymer-assisted intratumoral delivery of ethanol: Preclinical investigation of safety and efficacy in a murine breast cancer model
Source: PLoS One. 2021 Jan 28;16(1):e0234535. doi: 10.1371/journal.pone.0234535 (PMC7843014; doi:10.1371/journal.pone.0234535)
Supplement: S1 Fig — The methodology used to quantify the diffusion coefficient of ethanol in the tumor (DT). The experimental setup used for the Raman spectroscopy assay (left), consists of a tumor laying on a fully permeable membrane that allows seamless diffusion of ethanol upwards into the tumor. The CRS instrument captures the concentration of ethanol at the surface of the tumor over time. A deterministic transport model (right) characterizes ethanol transport and enables optimization of DT via fitting between the Raman measurements and the theoretical predictions. (PDF) [file pone.0234535.s002.pdf]

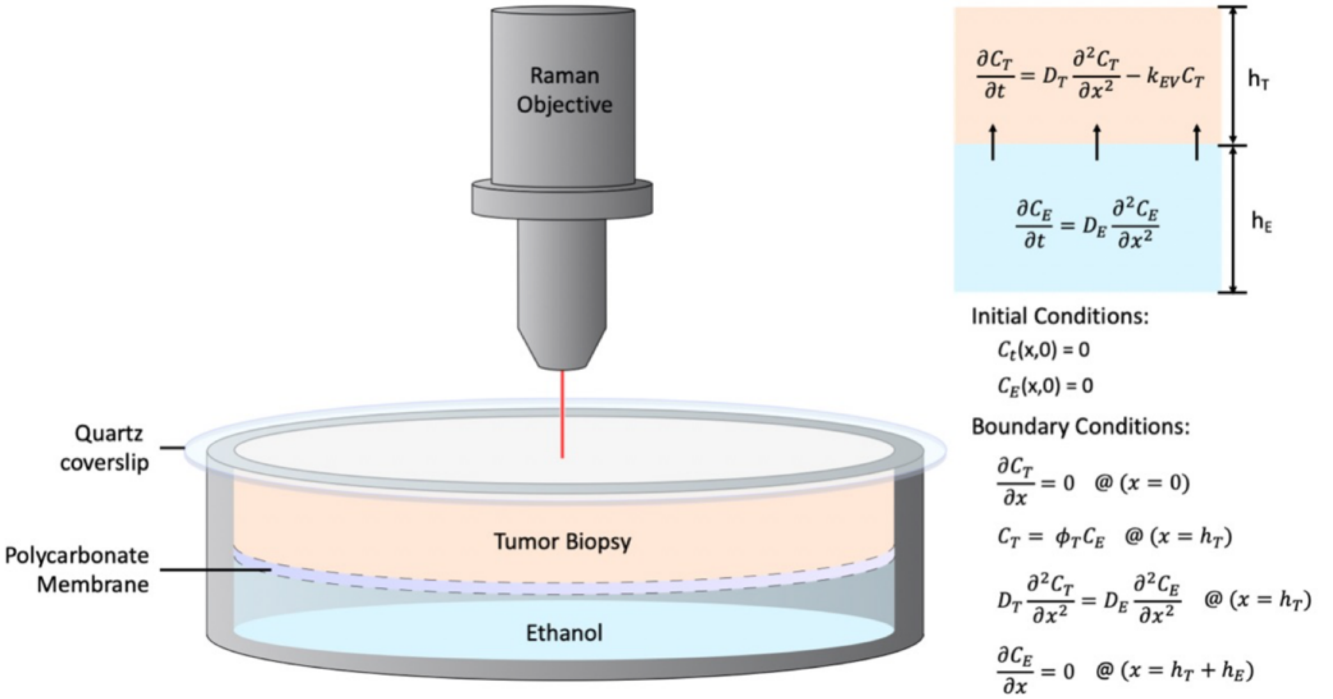
Raman Objective
Quartz coverslip
Tumor Biopsy
Polycarbonate Membrane
Ethanol
$\frac{\partial C_T}{\partial t} = D_T \frac{\partial^2 C_T}{\partial x^2} - k_{EV} C_T$
h_T
$\frac{\partial C_E}{\partial t} = D_E \frac{\partial^2 C_E}{\partial x^2}$
h_E
Initial Conditions:
$C_t(x,0) = 0$
$C_E(x,0) = 0$
Boundary Conditions:
$\frac{\partial C_T}{\partial x} = 0 \quad @ \, (x = 0)$
$C_T = \phi_T C_E \quad @ \, (x = h_T)$
$D_T \frac{\partial^2 C_T}{\partial x^2} = D_E \frac{\partial^2 C_E}{\partial x^2} \quad @ \, (x = h_T)$
$\frac{\partial C_E}{\partial x} = 0 \quad @ \, (x = h_T + h_E)$
