# Supplementary figures and images for: Polymer-assisted intratumoral delivery of ethanol: Preclinical investigation of safety and efficacy in a murine breast cancer model
Source: PLoS One. 2021 Jan 28;16(1):e0234535. doi: 10.1371/journal.pone.0234535 (PMC7843014; doi:10.1371/journal.pone.0234535)

**A**

EC-Ethanol

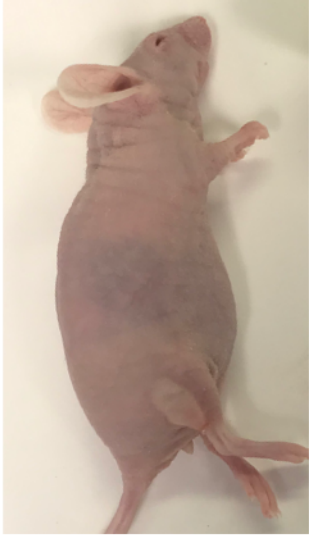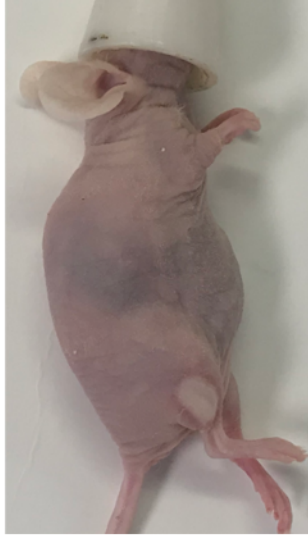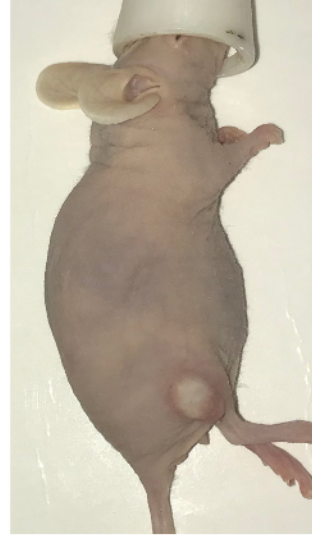

**B**

Ethanol

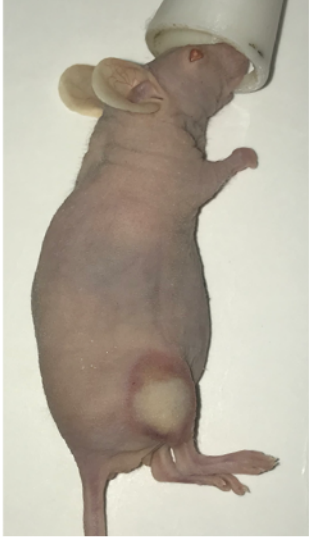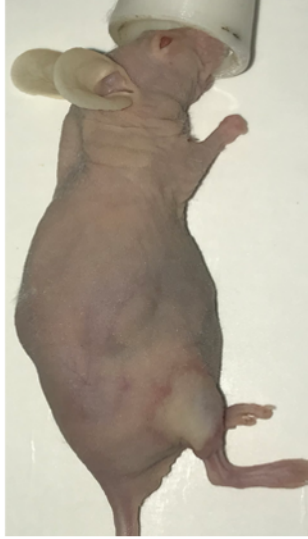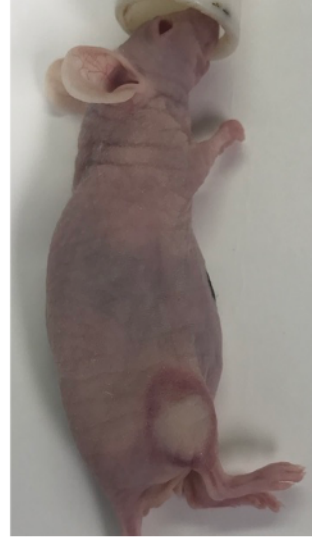

Supplement: S2 Fig — Mice after 100 μL injection of either A) EC-ethanol or B) ethanol alone into flank tumors. Nude mice used to show blanching and redness. Images taken approximately 5 minutes after injection. (PDF) [file pone.0234535.s003.pdf]

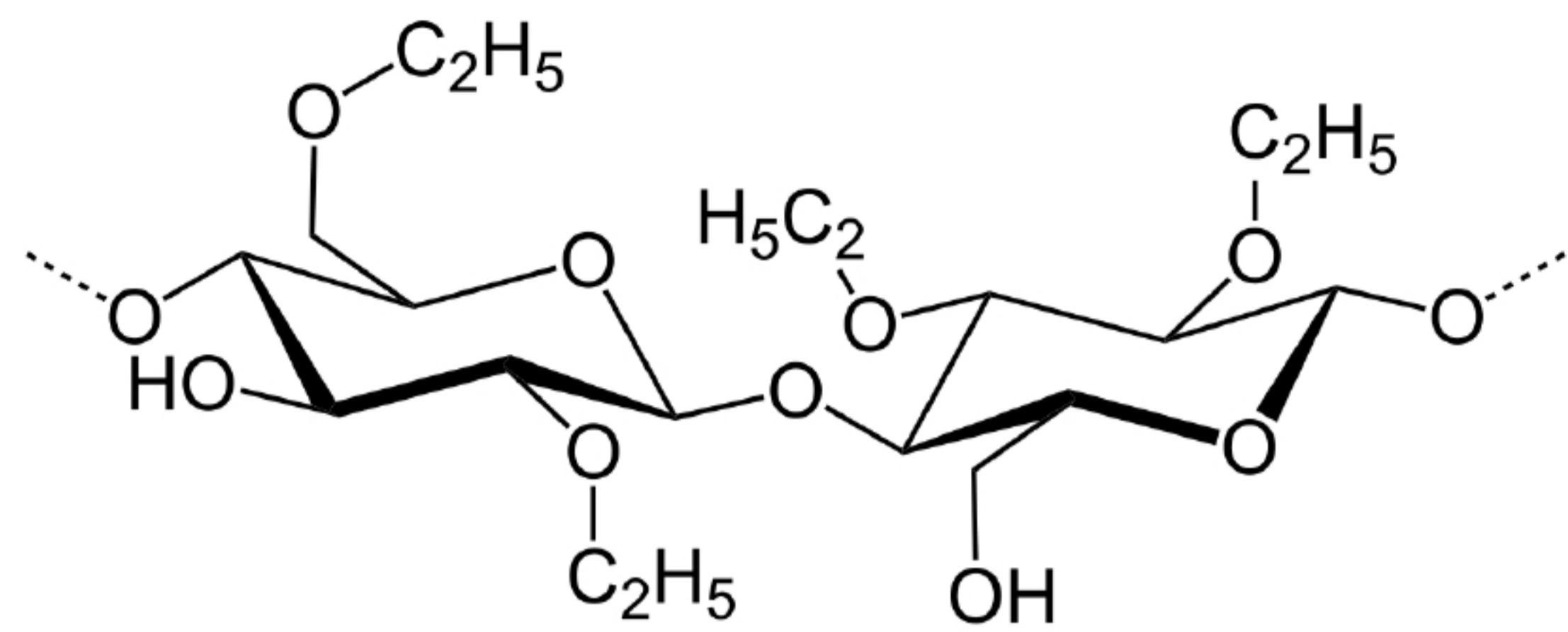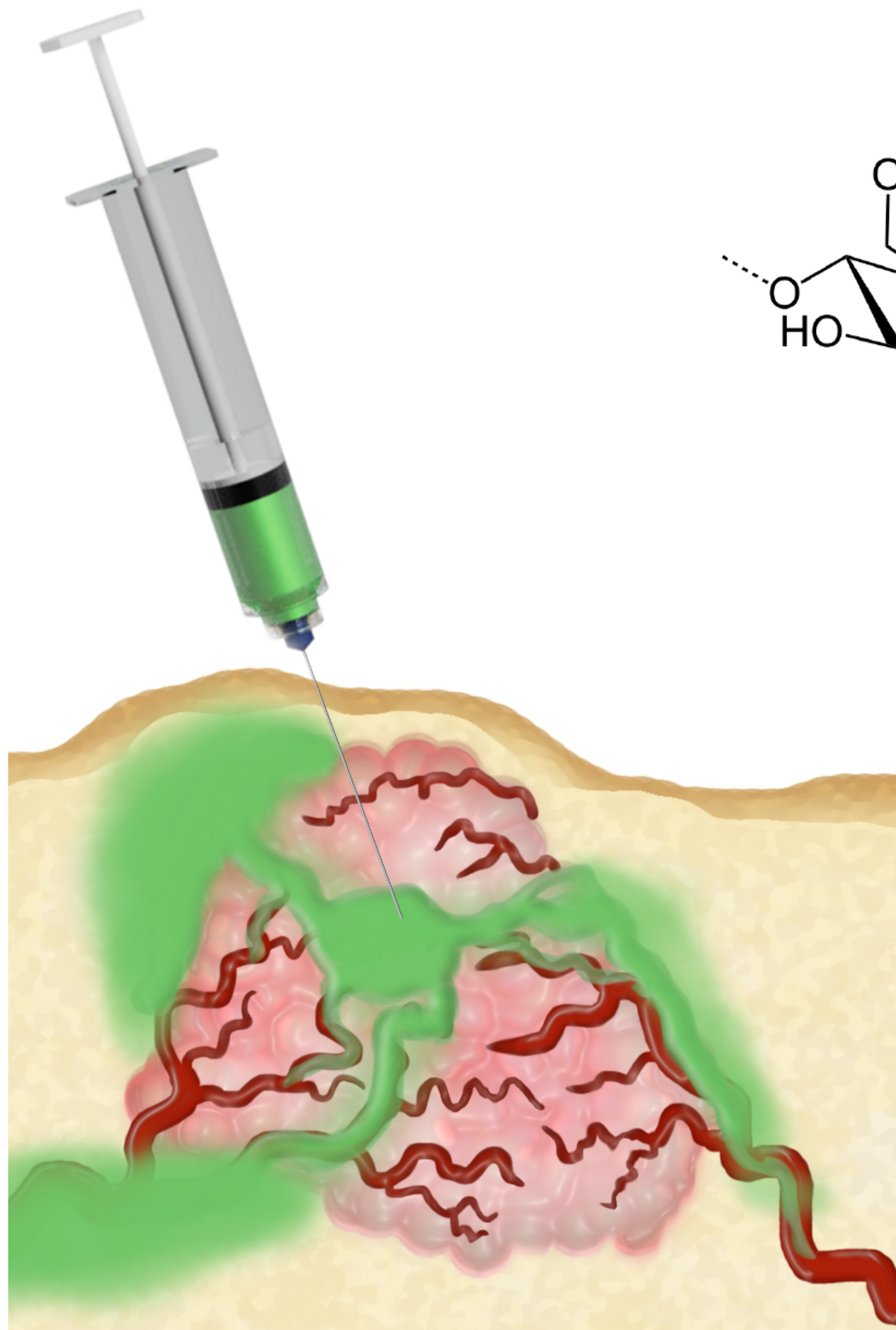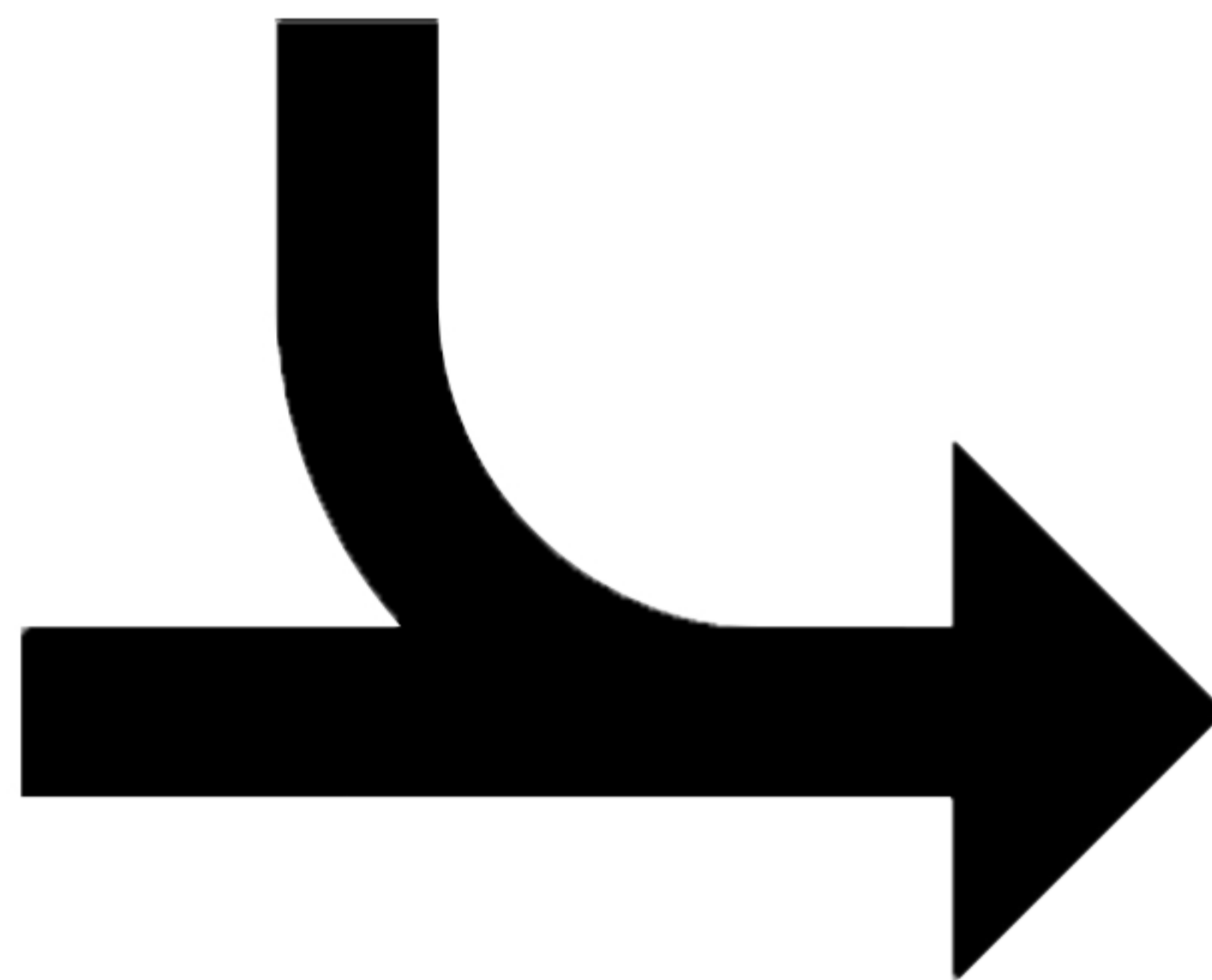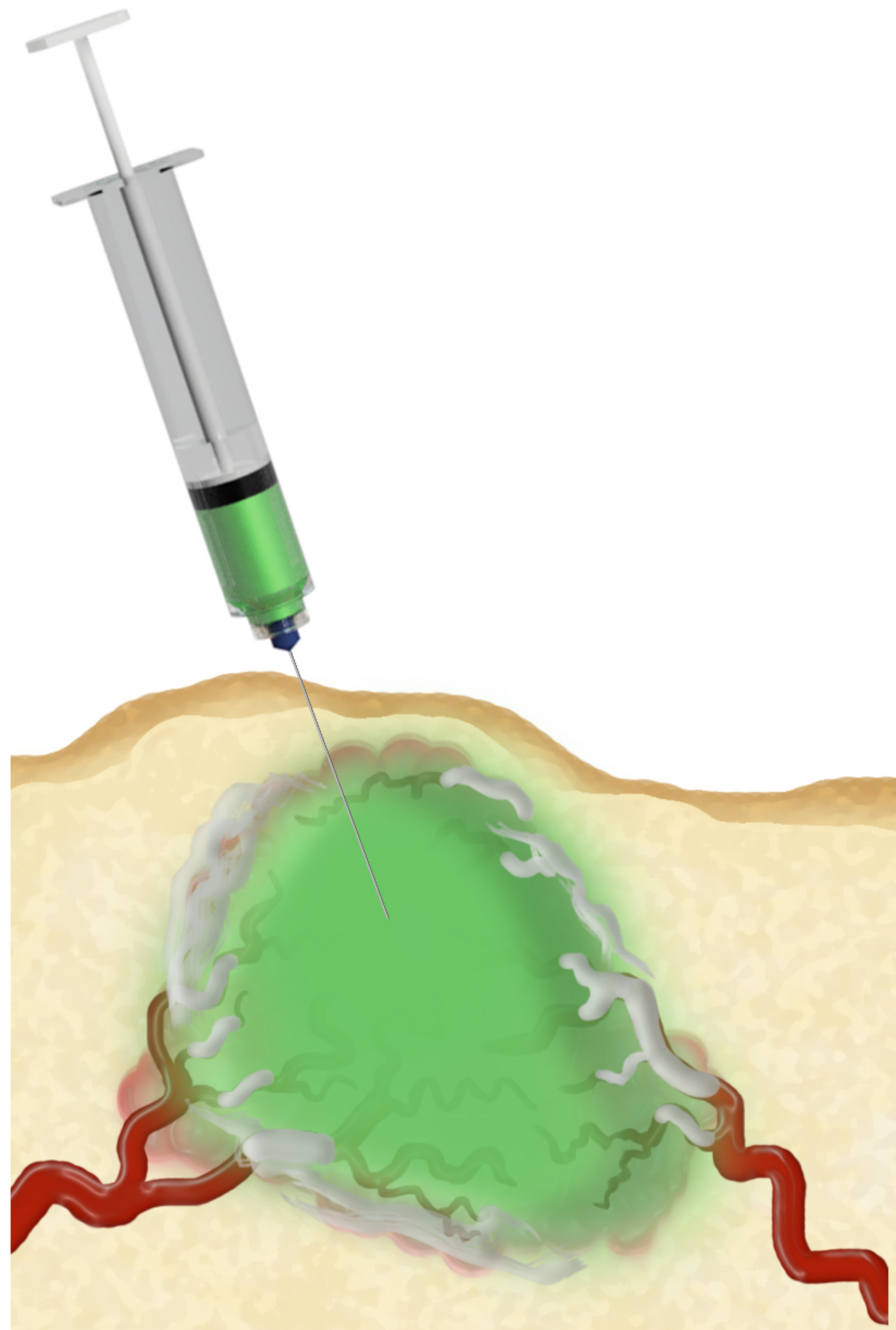

Supplement: S1 Graphical Abstract — (PDF) [file pone.0234535.s004.pdf]
